# Generation of macrophages with altered viral sensitivity from genome-edited rhesus macaque iPSCs to model human disease

**DOI:** 10.1016/j.omtm.2021.03.008

**Published:** 2021-03-17

**Authors:** Yoshihiro Iwamoto, Yohei Seki, Kahoru Taya, Masahiro Tanaka, Shoichi Iriguchi, Yasuyuki Miyake, Emi E. Nakayama, Tomoyuki Miura, Tatsuo Shioda, Hirofumi Akari, Akifumi Takaori-Kondo, Shin Kaneko

**Affiliations:** 1 Shin Kaneko Laboratory, Department of Cell Growth and Development, Center for iPS Cell Research and Application (CiRA), Kyoto University, Kyoto, Japan; 2 Department of Hematology and Oncology, Graduate School of Medicine, Kyoto University, Kyoto, Japan; 3 Center for Human Evolution Modeling Research, Primate Research Institute, Kyoto University, Kyoto, Japan; 4 Research Institute for Microbial Diseases, Osaka University, Osaka, Japan; 5 Laboratory of Primate Model, Research Center for Infectious Diseases, Institute for Frontier Life and Medical Science, Kyoto University, Kyoto, Japan; 6 Laboratory of Infectious Disease Model, Institute for Frontier Life and Medical Sciences, Kyoto University, Kyoto, Japan

**Keywords:** non-human primate, iPSC, HIV, macrophage, genome editing, CRISPR/Cas9

## Abstract

Because of their close biological similarity to humans, non-human primate (NHP) models are very useful for the development of induced pluripotent stem cell (iPSC)-based cell and regenerative organ transplantation therapies. However, knowledge on the establishment, differentiation, and genetic modification of NHP-iPSCs, especially rhesus macaque iPSCs, is limited. We succeeded in establishing iPSCs from the peripheral blood of rhesus macaques (Rh-iPSCs) by combining the Yamanaka reprograming factors and two inhibitors (GSK-3 inhibitor [CHIR 99021] and MEK1/2 inhibitor [PD0325901]) and differentiated the cells into functional macrophages through hematopoietic progenitor cells. To confirm feasibility of the Rh-iPSC-derived macrophages as a platform for bioassays to model diseases, we knocked out *TRIM5* gene in Rh-iPSCs by CRISPR-Cas9, which is a species-specific HIV resistance factor. *TRIM5* knockout (KO) iPSCs had the same differentiation potential to macrophages as did Rh-iPSCs, but the differentiated macrophages showed a gain of sensitivity to HIV infection *in vitro*. Our reprogramming, gene editing, and differentiation protocols used to obtain Rh-iPSC-derived macrophages can be applied to other gene mutations, expanding the number of NHP gene therapy models.

## Graphical abstract

**Figure:**
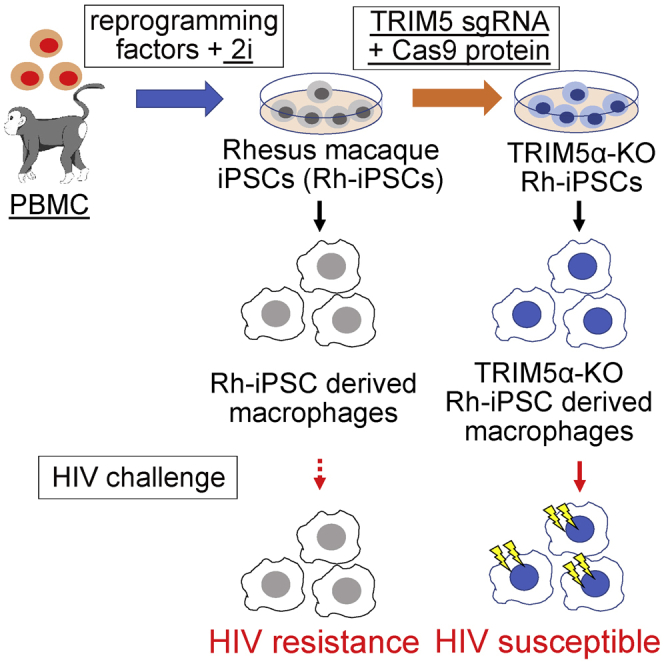

## Teaser

Non-human primate (NHP) models are thought to be very useful for the safety and efficacy evaluation of iPSC-based cell therapy. As a primary step for creating preclinical NHP models, Iwamoto and colleagues develop methods for reprogramming, differentiation, and gene editing techniques with CRISPR-Cas9 for rhesus macaque cells.

## Introduction

Induced pluripotent stem cells (iPSCs) are expected to have many clinical applications in regenerative medicine because of their unlimited self-renewal ability and potential to differentiate into any type of cell or tissue.^1^ Several groups, including ours, are preparing iPSCs from mature blood cells and differentiating them into hematopoietic stem cells, lymphocytes, and macrophages with the aim of treating a wide variety of diseases, including cancer and viral infections.^2^, ^3^, ^4^ In addition, iPSCs are an ideal platform to perform genetic engineering such as genome editing technology and viral gene transduction, because the genomic integrity of the edited cells can be thoroughly assessed due to their high cloning efficiency. The utility of iPSC-based regenerative medicine is further augmented when combined with gene engineering. In fact, the possibility of HIV treatment with macrophages derived from iPSCs transfected with short hairpin RNA (shRNA) targeting the HIV promotor has been reported.^5^ Functional immune cells induced by genome editing at the iPSC stage have also been reported.^6^, ^7^, ^8^

The *in vivo* evaluation of the efficacy and safety, including tumorigenicity and immunogenicity in preclinical models, is essential for the clinical application of iPSC products, but most studies have evaluated safety in immunodeficient mice only. Non-human primate (NHP) models are preferred preclinical animal models because of the stronger similarities between NHPs and humans compared with mice and humans. Accordingly, NHP-iPSCs for the treatment of retinal disease, Parkinson’s disease, heart disease, and hereditary bone disease have been reported, as have their use for the production of myocardial and bone cells and myocardial allogeneic transplantation.^9^, ^10^, ^11^, ^12^, ^13^

NHPs are phylogenetically very close to humans in their size, lifespan, and immune system.^12^^,^^14^^,^^15^ Additionally, the adaptive and innate immune responses to antigens in NHPs are very similar to those in humans. Among NHPs, rhesus macaques (Rh) are suitable for immunological analysis, including studies investigating viral infections and allogeneic transplantations, because their major histocompatibility complexes (MHCs) have been analyzed in detail.^16^, ^17^, ^18^ Accordingly, iPSCs of reprogrammed Rh cells (Rh-iPSCs) and Rh-iPSC-derived immune cells may be useful tools for studying immune responses *in vitro* and *in vivo*. In addition, Rh-iPSCs resemble human iPSCs in terms of morphology, marker expression, and growth factor dependency.^19^

In this study, as a proof of concept for the gain or loss of function in cells differentiated from gene-edited Rh-iPSCs, we knocked out *TRIM5* in Rh-iPSCs by the CRISPR-Cas9 system. TRIM5α is known to be a species-specific HIV resistance factor in Rh.^20^, ^21^, ^22^ It is also reported that the HIV resistance of Rh CD4 lymphocytes is lost *in vitro* by the knockout (KO) of TRIM5α in peripheral blood CD4 lymphocytes with TALEN.^23^ We differentiated wild-type and *TRIM5* KO Rh-iPSCs into hematopoietic progenitor cells (HPCs) and macrophages and compared their functions, finding that the KO iPSC products showed a gain of sensitivity to HIV.

The reprogramming, differentiation, and gene editing techniques for Rh cells presented in this paper will contribute to the development of preclinical NHP models for HIV infection using CCR5 KO HPC transplantation^24^^,^^25^ or allogeneic organ transplantations derived from histocompatibility leukocyte antigen (HLA)-KO iPSCs.^26^

## Results

### Generation of Rh-iPSCs from Rh peripheral blood mononuclear cells

Rh-iPSCs have been established from fibroblasts, bone marrow stromal cells, and CD34^+^ hematopoietic stem/progenitor cells (HSPCs),^11^^,^^12^^,^^19^^,^^27^, ^28^, ^29^ but not from peripheral blood mononuclear cells (PBMCs), which has become mainstream from the viewpoints of invasiveness, sterility, and ease of collection for generation of human iPSCs.^2^^,^^3^^,^^7^

**Figure 1:**
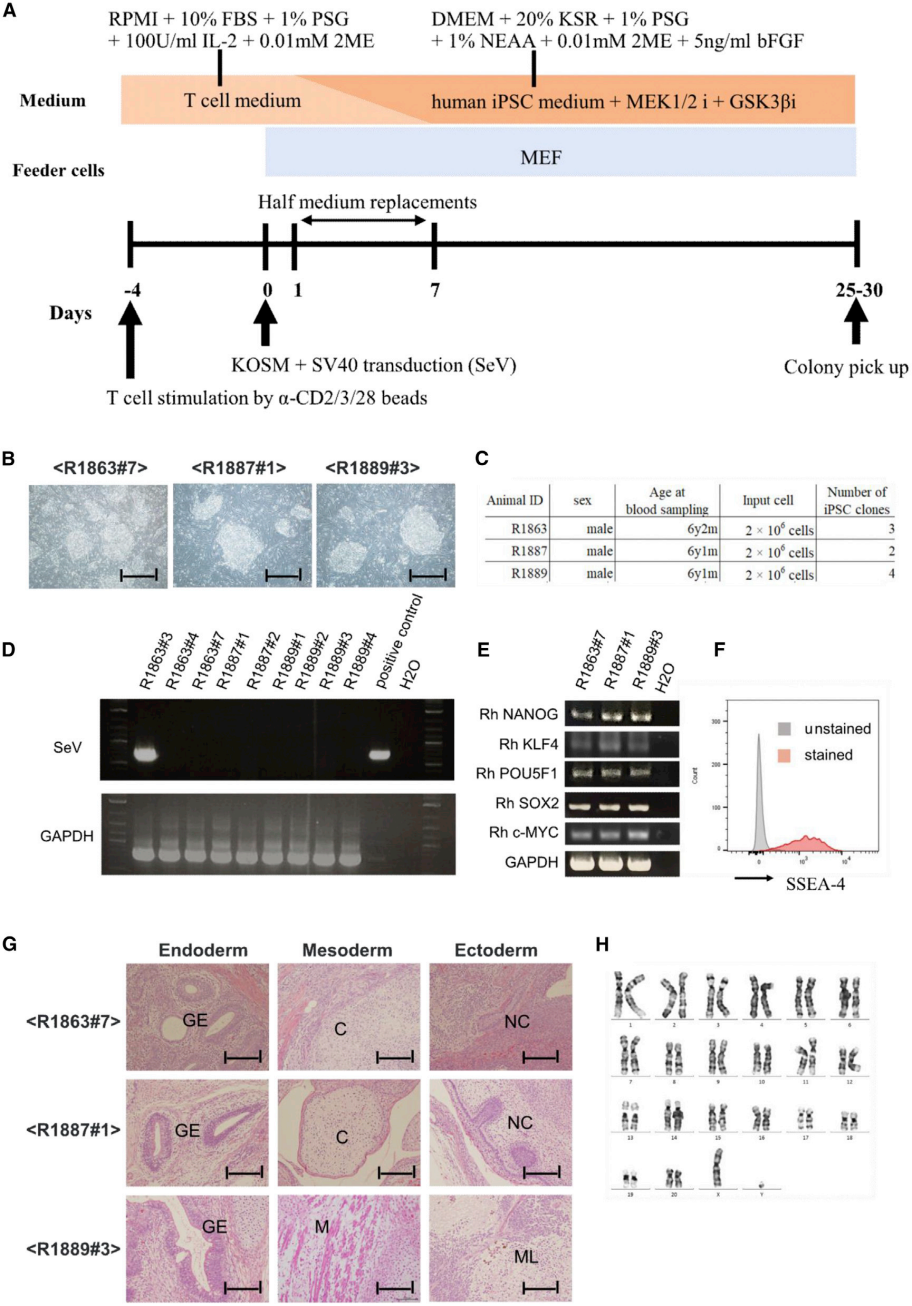
Generation of Rh-iPSCs from Rh PBMCs (A) Schematic illustration showing the reprogramming of PBMCs to Rh-iPSCs. (B) Phase contrast images of iPSC colonies from three individuals. Scale bars, 200 μm. (C) Summary of the Rh-iPSC generation. (D) RT-PCR analysis of SeV vectors. GAPDH was examined as an internal control. (E) RT-PCR analysis of pluripotency-associated genes. GAPDH was examined as an internal control. (F) Flow cytometric analysis of SSEA-4 expression. (G) Teratoma formation assay shows derivatives of all three germ layers. GE, gut-like epithelium; C, cartilage; M, muscle tissue; NC, neural crest; ML, melanocytes. Scale bars, 50 μm. (H) Chromosomal analysis of Rh-iPSCs.

In this study, following the reprogramming protocol for human iPSCs, we transfected Rh PBMCs with a Sendai virus (SeV) vector encoding the Yamanaka factors (OCT3/4, SOX2, KLF4, and c-MYC)^30^ to establish Rh-iPSCs. Notably, no colonies were observed when medium including basic fibroblast growth factor (bFGF), which is typically used to reprogram Rh fibroblasts, was used for the reprogramming. Therefore, we applied 2i medium, which adds GSK-3 inhibitor (CHIR 99021) and MEK1/2 inhibitor (PD0325901)^31^ to the original iPSC maintenance medium. In this case, dome-shaped colonies were observed about 25–30 days after the transfection (Figures 1A and 1B). The reprogramming was confirmed using cells from three Rh (animal IDs: R1863, R1887, R1889) (Figure 1C). Residual SeV was detected in one Rh-iPSC clone prepared from one individual, but not in any of the remaining clones (Figure 1D). We confirmed that the Rh-iPSCs expressed Nanog, KLF4, POU5F1, SOX2, and c-Myc by RT-PCR, and SSEA4, a marker of undifferentiated iPSCs, by fluorescence-activated cell sorting (FACS) (Figures 1E and 1F). We also verified pluripotency by detecting teratoma that could differentiate into the three germ layers (Figure 1G). Finally, Rh-iPSCs could be maintained for more than 50 passages with normal karyotype (Figure 1H).

### Differentiation of Rh-iPSCs into HPCs

**Figure 2:**
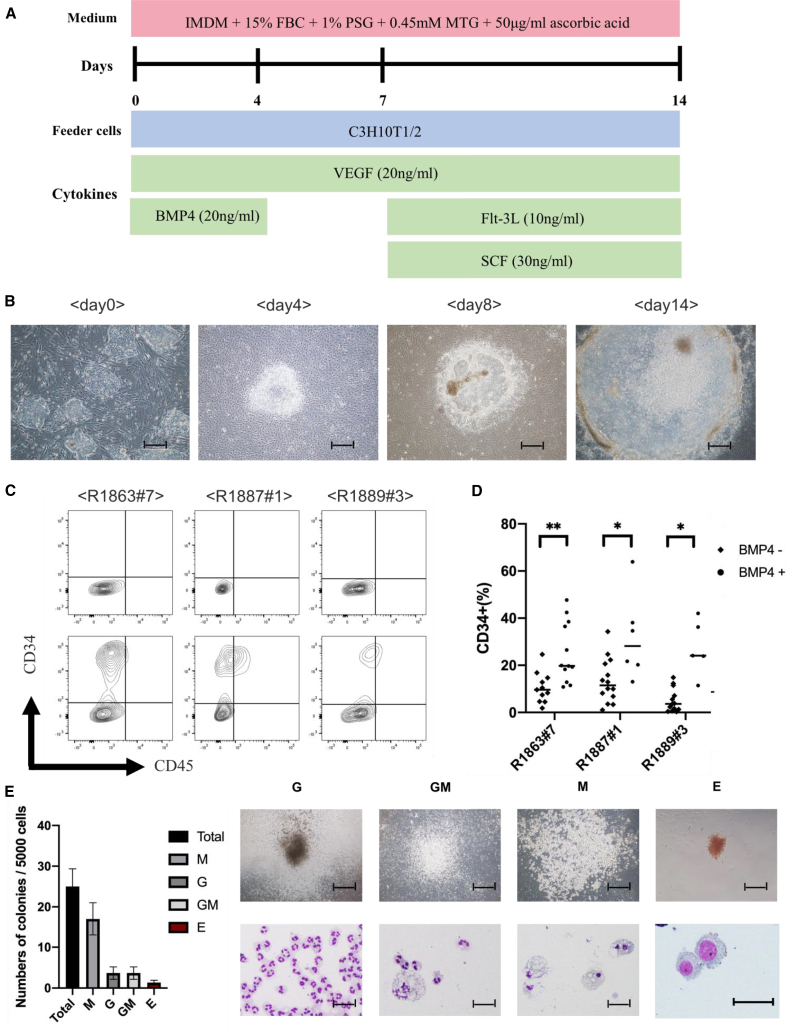
Differentiation of Rh-iPSCs into HPCs (A) Schematic illustration showing Rh-iPSC differentiation to HPCs. (B) Phase contrast images of Sac differentiation on days 0, 4, 8, and 14. Scale bars, 200 μm. (C) Flow cytometric analysis of the HPC phenotypes 14 days after starting the differentiation. Upper panels, unstained cells; lower panels, stained cells. (D) Dot plots show the differentiation efficiency into CD34^+^ cells of three Rh-iPSC clones with or without BMP4. ∗p < 0.05; ∗∗p < 0.01. (E) Bar graph displaying the number of colony-forming units. Data are plotted as the mean ± SD of triplicate samples. The microscopic images show colony morphology (upper panels) and cytospins (lower panels). G, granulocytes; M, macrophages; GM, granulocytes/macrophages; E, erythroid. Scale bars, 100 μm (top) and 50 μm (bottom).

By applying a reported human iPSC differentiation protocol^2^^,^^5^ (Figure 2A), we could differentiate all Rh-iPSC clones into CD34^+^ cells (Figures 2B and 2C). To improve the efficiency of the CD34^+^ cell induction, we added BMP4, which is reported to promote mesoderm differentiation,^32^, ^33^, ^34^ on day 0 of the protocol (Figure 2D). Table 1 shows a summary of the number of CD34^+^ cells. A colony-forming unit (CFU) assay was performed to evaluate whether the CD34^+^ cells represent HPCs. We confirmed colonies containing CFU-M (macrophage), CFU-GM (granulocyte-macrophage), CFU-G (granulocyte), and CFU-E (erythroid) at an efficiency of about 0.6% (Figure 2E). These results indicate that CD34^+^ cells differentiated from Rh-iPSCs were multipotential HPCs.

**Table 1:** Summary of CD34^+^ cells from Rh-iPSCs (confluent in 6-cm dish)

	Total cells (×10^6^)	CD34^+^ (%)	CD34^+^ cells (×10^6^)
R1863#7	8.69 ± 0.107	31.467 ± 5.147	2.63 ± 0.009

R1887#1	15.07 ± 0.30	25.567 ± 3.1	3.92 ± 0.110

R1889#3	18.91 ± 0 0.856	23.267 ± 6.716	3.48 ± 0.068

Data shown are mean ± SE from three independent experiments.

### Differentiation of Rh-iPSCs into macrophages

**Figure 3:**
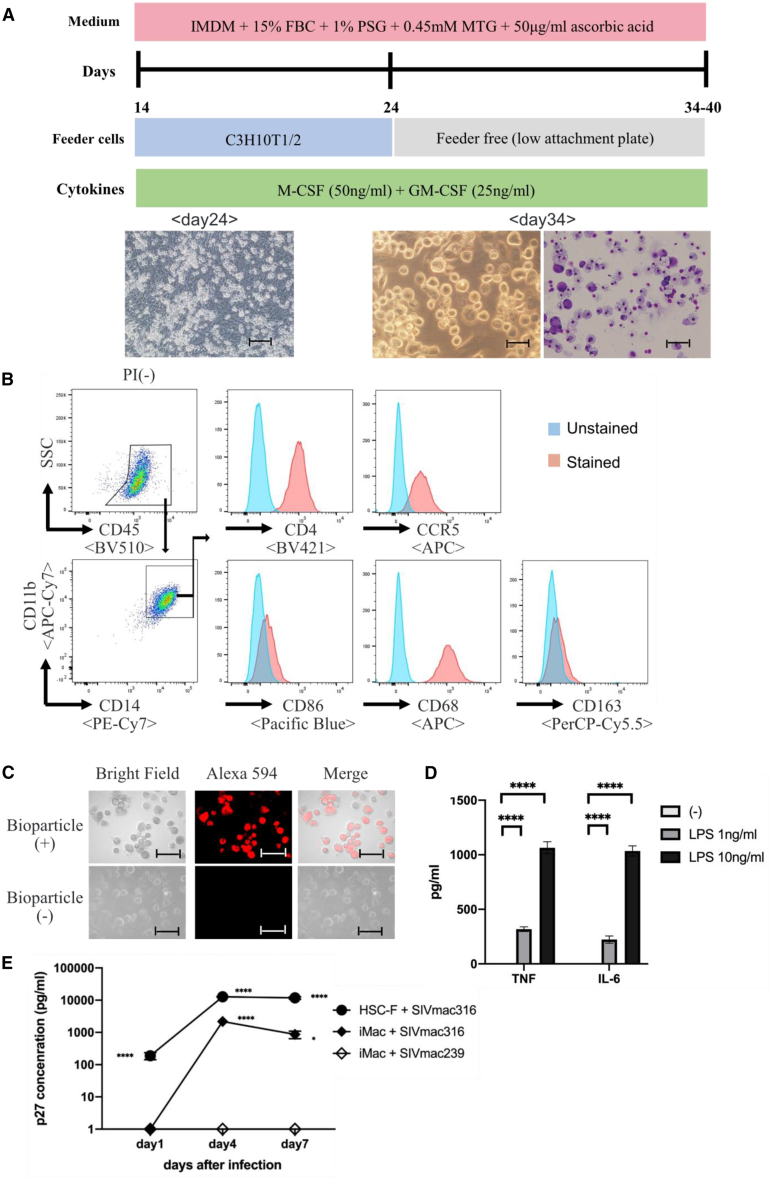
Differentiation of Rh-iPSCs into macrophages (A) Schematic illustration of the differentiation into macrophages from HPCs. Phase contrast images of macrophage differentiation on days 24 and 34 (left and middle, respectively) and cytospins on day 34 (right). Scale bars, 50 μm. (B) Flow cytometric analysis of the macrophage phenotypes 34 days after the differentiation. (C) Macrophages were incubated with Alexa Fluor 594 *Escherichia coli* and observed 1 h later. Microscopic images show bioparticles localized in the macrophages. Scale bars, 100 μm. (D) Cytokine production by macrophages differentiated from Rh-iPSCs. Data are plotted as the mean ± SD of triplicate samples. ∗∗∗∗p < 0.0001. (E) Detection of SIVmac316 in iPSC-derived macrophages by ELISA. HSC-F is a cynomolgus monkey T cell line and control. Replication was monitored by determining the amount of p27 in the culture supernatant at days 1, 4, and 7 after the incubation. Data are plotted as the mean ± SD of triplicate samples. ∗p < 0.05, ∗∗∗∗p < 0.0001 for comparisons between SIV mac 316 and SIV mac 239. iMac, iPSC-derived macrophage.

Next, we induced Rh-iPSC-derived HPCs into macrophages, which are target cells for HIV/SIV (simian immunodeficient virus), using the human iPSC differentiation method established by our group.^5^ After the HPCs were co-cultured with C3H10T1/2 feeder for 10 days in the presence of macrophage colony-stimulating factor (M-CSF) and granulocyte-macrophage CSF (GM-CSF), adherent cells were seeded on a low-adsorption plate and further cultured for about 10 days to differentiate into macrophages (Figure 3A). Starting with confluent Rh-iPSCs in a 6-cm dish, about 1–2 × 10^7^ macrophages could be obtained on day 34. After day 34, the macrophages showed no obvious growth. FACS analysis showed that the induced macrophages were CD11b^+^/CD14^+^/CD68^+^/CD86^+^/CD163^−^. They also expressed CCR5, which is a co-receptor for HIV/SIV (Figures 3B and S1A). The phenotypes of the Rh-iPSC-derived macrophages resembled those of monocyte-derived macrophages (Figure S1B). We confirmed phagocytosis by the induced macrophages after 1 h of co-culturing with Alexa Fluor 594-conjugated *Escherichia coli* bioparticles (Figure 3C). Additionally, macrophages stimulated with lipopolysaccharide (LPS) produced the inflammatory cytokines tumor necrosis factor (TNF) and interleukin (IL)-6 (Figure 3D).

We also stimulated the induced macrophages by SIV infection. Macrophage tropic SIVmac316 and T cell tropic SIVmac239 were co-cultured with the induced macrophages, and p27 protein was measured by ELISA on days 1, 4, and 7. The production of p27 protein was observed in the SIVmac316 co-culture but not in the SIVmac239 co-culture (Figure 3A). These results suggest that functional macrophages with SIV sensitivity can be differentiated from Rh-iPSCs by our protocol.

### Generation of TRIM5 KO Rh-iPSCs by CRISPR-Cas9

**Figure 4:**
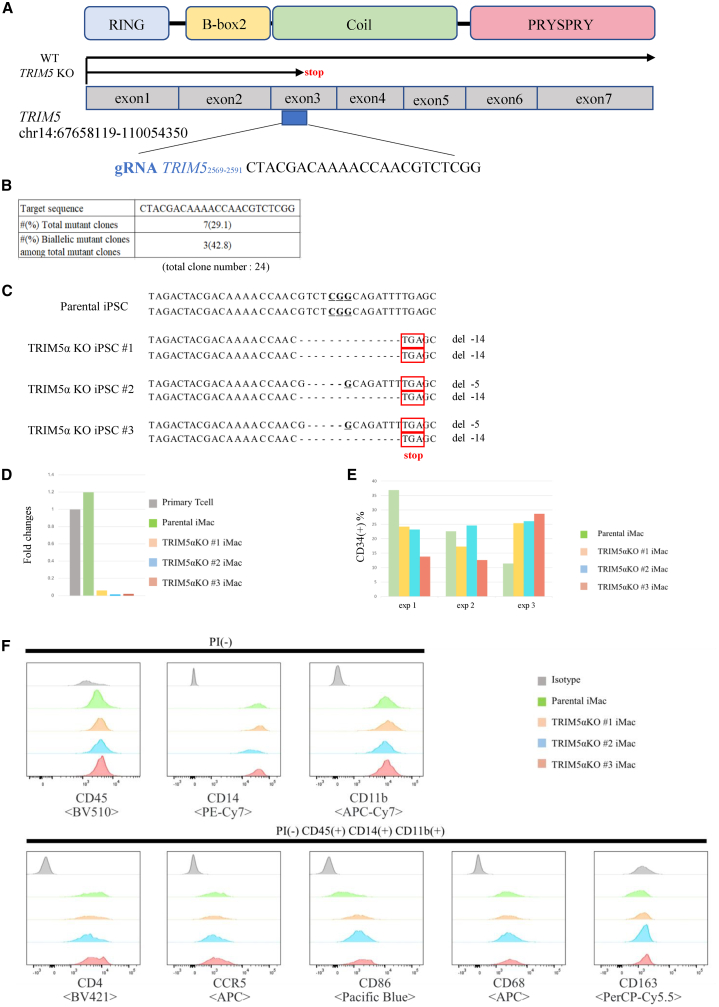
Generation of TRIM5αKO Rh-iPSCs (A) Schematic illustration of the sgRNA target site in the Rh *TRIM5* gene. (B) sgRNA target sequence and efficiency of the genome editing. (C) Sequence data of the TRIM5α homo-KO iPSC clones and parental iPSC clone. Three established clones had biallelic frameshift mutations in the *TRIM5* gene at the indicated sites. (D) Rh *TRIM5* expression in TRIM5α homo-KO iPSC clones and parental iPSC clone was evaluated with real-time PCR. Fold changes relative to primary Rh T cells are shown. (E) The differentiation efficiency to CD34^+^ cells of parental and TRIM5αKO iPSCs. Three independent experiments. (F) Flow cytometric analysis of macrophage-marker expression in macrophages generated from parental and TRIM5αKO iPSCs.

We next used CRISPR-Cas9 to genome edit the Rh-iPSCs. TRIM5α is an HIV resistance factor in Rh. It has a PRYSPRY region at its C-terminal that is connected via a long link to the N-terminal, which includes three motifs: a RING domain, B-box 2 domain, and coiled-coil domain. We knocked out TRIM5α using the CRISPR-Cas9 system in Rh-iPSCs. Candidate sequences for the single guide RNA (sgRNA) were selected using CRISPOR (http://crispor.tefor.net/crispor.py). The target sequence is shown in Figure 4A. We transfected sgRNA and Cas9 protein into Rh-iPSCs by electroporation and picked up 24 colonies manually without drug selection. An analysis of the genomic sequences of *TRIM5* in the 24 clones revealed mutations in 7 clones, with 3 of them showing homozygous mutations in *TRIM5* (Figure 4B). These clones had an in-frame mutation in a single allele. By randomly selecting one clone from the heterozygous clones and performing additional genome editing, we were able to create TRIM5αKO Rh-iPSCs, in which the stop codon due to a frameshift mutation was confirmed (Figure 4C). The PRYSPRY region is considered to be important for controlling HIV infection.^21^ Because the stop codon existed at exon3 in TRIM5αKO Rh-iPSCs, the PRYSPRY region downstream of exon3 was not translated. Thus, we expected TRIM5αKO Rh-iPSCs to have lost their resistance to HIV infection. When the expression of TRIM5αKO Rh-iPSC mRNA was measured by qPCR, the expression level of TRIM5α was decreased in all three strains, which is considered to be caused by nonsense mutation-dependent mRNA degradation (NMD)^35^^,^^36^ (Figure 4D). Next, we evaluated the differentiation potential of TRIM5αKO Rh-iPSCs in reference to parental Rh-iPSCs. Parental Rh-iPSCs and TRIM5αKO Rh-iPSCs had equivalent efficiencies for differentiating into CD34^+^ cells and macrophages (Figures 4E and 4F). The expression levels of CD86 were different between macrophages differentiated from parental and TRIM5αKO. One report found that primate dendritic cells (DCs) lacking efficient TRIM5α-mediated retroviral restriction upregulate CD86 expression.^37^ We hypothesize that the expression of CD86 was increased in TRIM5αKO macrophages by the same mechanism. Additionally, we confirmed no potential off-target sites in the TRIM5αKO Rh-iPSC clones for the guide RNAs (gRNAs), as identified by CRISPOR software and Sanger sequencing (Table 2). From these results, we confirmed that the genome editing of Rh-iPSCs did not compromise the differentiation potential.

**Table 2:** Results of off-target sequence analysis of top five off-target candidates determined by the CFD score of TRIM5 gRNA

	Position	Sequence (5′→3′)	No. of mismatches	CFD off-target score	KO iPSC #1	KO iPSC #2	KO iPSC #3
TRIM5 on-target site	chr14:67658119–110054350	CTACGACAAAACCAACGTCT CGG	–	1.0000	−14 bp, −14 bp	−5 bp, −14 bp	−14 bp, −14 bp

Potential off-target sites	chr4:103904324–103904346	CTATAATAAAACCAACATCT TGG	4	0.525778	WT	WT	WT
	chr2:76073316–76073338	CTAAGGCAAAAACAACATCT AGG	4	0.401003	WT	WT	WT
	chr2:98763243–98763265	CTGCCACAAACCCAACATCT TGG	4	0.179259	WT	WT	WT
	chr7:42594298–42594320	ATAAGATAAAACCAACGTCT GAG	3	0.177388	WT	WT	WT
	chr9:104127964–104127986	CTACAAAGAAACCAACTTCT AGG	4	0.119167	WT	WT	WT

CFD, cutting frequency determination; WT, wild-type.

### Deletion of TRIM5α enables HIV-1 virus infection

**Figure 5:**
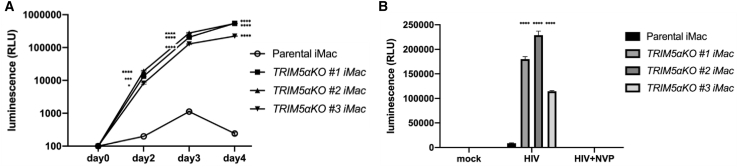
Deletion of TRIM5α enables HIV-1 virus infection (A) Significant increase of HIV luminescence of TRIM5αKO iMacs compared to parental iMacs. Macrophages (1 × 10^5^ cells/well) were infected with NL43-Luci/VSV-G (10 ng of p24 virus) in a 24-well plate. Luciferase activity was measured 2, 3, and 4 days after the infection. Data are plotted as the mean ± SD of triplicate samples and presented as three independent measurements. ∗p < 0.05, ∗∗∗p < 0.001, ∗∗∗∗p < 0.0001 for comparisons between TRIM5αKO iMacs and parental iMacs. (B) NL43-Luci/VSV-G infection was inhibited by nevirapine (NVP). Macrophages (5 × 10^4^ cells/well) were infected with NL43-Luci/VSV-G (6.6 ng of p24 virus) in a 96-well plate. Luciferase activity was measured 2 days after the infection. Data are plotted as the mean ± SD of triplicate samples and presented as three independent measurements of a single experiment. ∗∗∗∗p < 0.0001 for comparisons between TRIM5αKO iMacs and parental iMacs.

In order to confirm that TRIM5α was functionally knocked out in TRIM5αKO Rh-iPSCs, we infected the induced macrophages with HIV (vesicular stomatitis virus glycoprotein G [VSV-G]-pseudotyped lentivirus vector expressing luciferase [NL43-Luci/VSV-G]^38^). HIV (NL43-Luci/VSV-G) was co-cultured with macrophages differentiated from TRIM5αKO Rh-iPSCs and parental Rh-iPSCs. Luminescence was increased in macrophages differentiated from TRIM5αKO Rh-iPSCs compared with parental Rh-iPSCs on all days observed (2, 3, and 4), suggesting that the TRIM5αKO Rh-iPSC-derived macrophages lost their resistance to the early stage of HIV infection (Figure 5A). To determine whether the HIV infection was caused by the loss of TRIM5α, which degrades HIV at the reverse transcription stage, we tested a reverse transcriptase inhibitor (nevirapine [NVP]), finding that it suppressed the HIV infection of TRIM5αKO Rh-iPSC-derived macrophages (Figure 5B). From these results, we showed that it is possible to control the HIV resistance of Rh-iPSC-derived macrophages by gene editing *TRIM5*.

## Discussion

As the first step in creating an NHP model for evaluating the efficacy and safety of genome-edited iPSC-derived cells, we established a method to generate iPSCs from Rh PBMCs, induce their differentiation to HPCs/macrophages, and edit target genes using the CRISPR-Cas9 system. As a proof of concept, we showed that macrophages from iPSCs in which TRIM5α was deleted lost their resistance in the early stage of HIV infection.

By adding GSK-3 inhibitor (CHIR 99021) and MEK1/2 inhibitor (PD0325901) (i.e., 2i) to the iPSC medium, we could generate iPSCs from Rh PBMCs that can be collected aseptically and relatively easily. 2i was originally reported to maintain mouse embryonic stem cells (ESCs),^31^ but has since been applied to maintain iPSCs from various species.^39^, ^40^, ^41^ Interestingly, Rh-iPSCs have been established and maintained without 2i, but from fibroblasts, bone marrow stromal cells, and CD34^+^ HPCs, all of which are shallowly differentiated cells. We found that 2i was required for reprogramming more differentiated cells such as PBMCs.

In this study, we show that BMP4 improved the efficiency to induce HPCs from Rh-iPSCs. This effect is consistent with human iPSCs^33^ and pigtail macaque iPSCs.^34^ In order to acquire a massive amount of HPCs for transplantation experiments, it is essential to optimize the differentiation induction conditions. With our culture method, we could generate about 1–2 × 10^7^ HPCs from rh-iPSCs cultivated on a 6-cm dish (Table 1). This massive amount is expected to achieve long-term engraftment by autologous or allogeneic transplantation when supported by the appropriate environment such as niche and cytokines.

Macrophages are immune cells that play an important role in eliminating pathogens and dying cells. They also express CD4 and CCR5, which are HIV/SIV receptors, and act as HIV/SIV virus reservoirs. The possibility of treating HIV infection with macrophages regenerated from human iPSCs transfected with shRNA targeting the HIV-1 promotor or with CCR5 KO has been reported.^5^^,^^8^ By using genome-edited Rh-iPSC-derived macrophages, the findings of human iPSC-derived macrophages can be verified with an NHP model, advancing research on viral infections including HIV/SIV and corresponding treatments. Following this approach, we confirmed that HIV could infect TRIM5αKO iPSC-derived macrophages, not CD4^+^ T cells. A protocol to induce CD4^+^ T cells is for future work.

Gene transfer by homology-directed repair using the CRISPR-Cas9 system for Rh-iPSCs has been reported,^29^ but the present study is the first to report functional loss due to non-homologous end joining. We found poor efficiency for the genome editing of Rh-iPSCs by the conventional CRISPR-Cas9 system using plasmid DNA (Table S1). Furthermore, the technique was technically difficult. In contrast, the ribonucleoprotein (RNP) method we used raises the efficiency while simplifying the sgRNA synthesis.

Finally, our genome editing of TRIM5 in Rh-iPSCs is a proof of concept for other target genes. One possibility is a gene therapy model for HIV created by transplanting HPCs derived from CCR5 KO Rh-iPSCs. Furthermore, the present method will contribute to preclinical models such as the development of NHP models for the allogeneic transplantation of HLA-KO iPSC-derived cells/tissues.

## Materials and methods

### Animal use

All animals used in this study were housed and handled in accordance with protocols approved by the Primate Research Institute, Kyoto University (2016-C-5).

### Generation of Rh-iPSCs from Rh PBMCs

Rh-iPSCs were generated from Rh PBMCs. PBMCs were stimulated by anti-CD2/3/28-coated beads (Miltenyi Biotec, catalog no. 130-092-919). After 4 days, the PBMCs were transduced with SeV vectors harboring OCT3/4, KLF2, SOX2, c-MYC,^30^ and SV40 large T antigen, and then seeded onto inactivated mouse embryonic feeder cells (MEFs). The cultured medium was gradually replaced with Rh-iPSC medium (Dulbecco’s modified Eagle’s medium/Ham’s F-12 medium [Sigma] supplemented with 20% KO serum replacer [Thermo Fischer Scientific], 1% l-glutamine-penicillin-streptomycin solution [Sigma], 1% nonessential amino acids [Thermo Fischer Scientific], 10 mM 2-mercaptoethanol, and 5 ng/mL bFGF [Wako]), in addition to 3 μM GSK-3 inhibitor CHIR 99021 (Tocris) and 2 μM MEK1/2 inhibitor PD0325901 (Wako). The established iPSC clones were transfected with small interfering RNA L527^30^ using Lipofectamine RNAi Max (Invitrogen) to remove the SeV vectors from the cytoplasm.

### Rh-iPSC maintenance and passage

Rh-iPSC medium was replaced with 1 mL per 60-mm dish of dissociation solution consisting of 20 mL of 2.5% trypsin (Invitrogen), 40 mL of KO serum replacement (Invitrogen), and 2 mL of 100 mM CaCl_2_ to 138 mL of Dulbecco’s PBS (D-PBS)(−) (Nacalai, Japan), and the cells were then incubated at 37°C in a CO_2_ incubator for 5 min. After the incubation, MEFs and dissociation solution were removed, and 1 mL of Rh-iPSC culture medium was added. The colonies were broken up into small cell clumps by pipetting. About one-fifth of the cell suspension was transferred to a new MEF dish, although the split ratio may require adjustment depending on the iPSC line. The medium was replaced with fresh medium every day.

### Teratoma formation

Rh-iPSCs at a confluency of 70%–80% were harvested by using trypsin EDTA, and 2 × 10^6^ iPSCs were suspended with 100 μL of Matrigel and 100 μL of cold PBS. Rh-iPSCs were injected subcutaneously into 6-week-old female NOD.Cg-*Prkdc*^*scid*^ *Il2rg*^*tm1Wjl*^/SzJ (NSG) mice, and 8–10 weeks later, teratomas were dissected and fixed in formaldehyde.

### Differentiation of Rh-iPSCs into HPCs

To induce hematopoietic differentiation from Rh-iPSCs, we slightly modified a previously described human iPSC method.^2^ In brief, small clumps (<100 cells) of Rh-iPSCs maintained on MEFs were collected and co-cultured on C3H10T1/2 feeder cells in EB medium (Iscove’s modified Dulbecco’s medium [Sigma] containing 20% fetal bovine serum [FBS], 1% l-glutamine-penicillin-streptomycin solution [Sigma], 100× insulin-transferrin-selenium solution [Thermo Fisher Scientific], 450 μM monothioglycerol [Nacalai], 50 μg/mL ascorbic acid 2-phosphate [Nacalai]), in addition to 20 ng/mL VEGF (R&D Systems). On day 0, 20 ng/mL BMP4 (R&D Systems), and on days 7, 10, and 12, 30 ng/mL stem cell factor (SCF) (R&D Systems) and 10 ng/mL FLT-3L (PeproTech) were added to EB medium. Hematopoietic cells generated in iPSC sacs were collected on day 14.

### Differentiation of Rh-iPSCs into macrophages

On day 14, the collected hematopoietic cells were transferred onto newly prepared C3H10T1/2 feeder cells in EB medium containing 50 ng/mL M-CSF (PeproTech) and 25 ng/mL GM-CSF (PeproTech). On day 24, after the floating and loosely adherent cells were removed, firmly adherent cells were collected and transferred to low-attachment six-well culture plates (Corning Costar ultra-low attachment multiwell culture plates; Sigma-Aldrich) in EB medium containing GM-CSF (50 ng/mL) and M-CSF (25 ng/mL) and differentiated to macrophages after about 10 more days.

### Flow cytometry

Stained cell samples were analyzed using an LSRFortessa (BD Biosciences), and the data were processed using FlowJo (Tree Star). The following antibodies were used: allophycocyanin (APC)-CD34 (clone 563; BD Biosciences), Brilliant Violet 510 (BV510)-CD45 (clone D058-1283; BD Biosciences), Brilliant Violet 421 (BV421)-CD4 (clone OKT4; BioLegend), APC-Cy7-CD11b (clone M1/70; BioLegend), phycoerythrin (PE)/Cy7-CD14 (clone M5E2; BioLegend), Alexa Fluor 648-CD68 (clone KP1; Santa Cruz), Pacific Blue-CD86 (clone IT2.2; BioLegend), peridinin chlorophyll protein (PerCP)-Cy5.5-CD163 (clone GHI/61; BioLegend), APC-CCR5 (clone 3A9; BD Bioscience), and PE-SSEA4 (clone FAB1435P; R&D Systems).

### Colony-forming unit assay

CD34^+^ cells were sorted using a FACSAria II flow cytometer (BD Biosciences) from day 14 of the hematopoietic differentiation culturing. A total of 5,000 cells per 35-mm dish were seeded in MethoCult (H4435; STEMCELL Technologies) containing 1% l-glutamine-penicillin-streptomycin solution (Sigma) and cultured for 14 days in a 37°C incubator. Representative colonies were picked up and analyzed by microscopic morphology after Giemsa staining.

### Analysis of phagocytosis function of macrophages differentiated from Rh-iPSCs

Macrophages were co-cultured with Alexa Fluor 594-conjugated *Escherichia coli* bioparticles (Thermo Fisher Scientific) for 1 h, washed three times with PBS, and observed by an IX71 inverted microscope (Olympus).

### Analysis of cytokine production of macrophages differentiated from Rh-iPSCs

Macrophages were cultured in 96-well plates (5 × 10^4^ cells/200 μL EB medium containing GM-CSF [50 ng/mL] and M-CSF [25 ng/mL]) in the presence of LPS (0, 1, or 10 ng/mL). After 24 h of culture, the supernatant was collected, and the concentrations of TNF and IL-6 were measured by using a cytometric bead array (CBA) human inflammatory cytokine kit (BD Biosciences, catalog no. 551811).

### *TRIM5* targeted sgRNA construction

Candidate sequences targeting rhesus *TRIM5* were selected using CRISPOR (http://crispor.tefor.net/crispor.py). The designed gRNAs were synthesized by *in vitro* transcription using a MEGAshortscript kit (Thermo Fisher Scientific, catalog no. AM1354) according to the instruction manual.

### CRISPR-Cas9 genome editing experiments

Transfection was performed by using MaxCyte STx (MaxCyte). 1.5 × 10^6^ iPSCs were transfected with an RNP complex consisting of 10 μg of recombinant Cas9 (Integrated DNA Technologies, catalog no. 1074181) and 2.5 μg of *in vitro*-transcribed (IVT) gRNA in 50 μL of HyClone electroporation buffer. After electroporation, the cells were transferred to a MEF-coated plate in Rh-iPSC medium containing ROCK inhibitor Y-27632 for 3 days and then passaged to a new MEF-coated plate. Without drug selection, single colonies were manually picked up and cloned.

### SIV infection and viral quantification

A total of 1 × 10^5^ differentiated macrophages were infected with SIV mac 316 or SIV mac 239 for 2 h at 37°C, washed twice with PBS to remove the free virus, and cultured for 10 days. The culture supernatants were collected at days 1, 4, and 7 and measured for p27 antigen by ELISA according to the manufacturer’s instructions (ZeptoMetrix, catalog no. 0801201).

### HIV-1 infection and luciferase assay

For VSV-G-pseudotyped lentivirus vector expressing luciferase (NL43-Luci/VSV-G)^38^ preparation, human embryonic kidney cells (293T cells) were transfected with 15 mg of pNL4-3.Luc.R-E-plasmid and 5 mg of VSV-G-encoding plasmid, and viruses were harvested 48 h later. Differentiated macrophages were infected with NL43-Luci/VSV-G for 2 h in a 37°C incubator, washed twice with PBS to remove the free virus, and cultured for 4 days in the presence or absence of 2 μM of an anti-HIV drug, nevirapine (Sigma). At days 2, 3, and 4, luciferase activity in the cell lysate was measured by using a luciferase assay system (Promega, catalog no. E1500) and Lumat LB 9507 (Berthold Technologies).

### Statistical analysis

GraphPad Prism (GraphPad) was used for all statistical analyses. Paired or unpaired Student’s t tests or two-way ANOVAs with a Tukey’s multiple comparison test were performed to assess the statistical significance of differences between groups.

## Supplemental information

Document S1. Figure S1 and Table S1

Document S2. Article plus supplemental information
