# Supplementary material for: Generation of macrophages with altered viral sensitivity from genome-edited rhesus macaque iPSCs to model human disease
Source: Mol Ther Methods Clin Dev. 2021 Mar 17;21:262–73. doi: 10.1016/j.omtm.2021.03.008 (PMC8039773; doi:10.1016/j.omtm.2021.03.008)
Supplement: Document S1. Figure S1 and Table S1 [file mmc1.pdf]

OMTM, Volume *21*

Supplemental information

Generation of macrophages with altered viral sensitivity from genome-edited rhesus macaque iPSCs to model human disease

Yoshihiro Iwamoto, Yohei Seki, Kahoru Taya, Masahiro Tanaka, Shoichi Iriguchi, Yasuyuki Miyake, Emi E. Nakayama, Tomoyuki Miura, Tatsuo Shioda, Hirofumi Akari, Akifumi Takaori-Kondo, and Shin Kaneko

Figure S1

a

<u><R1863#7></u>

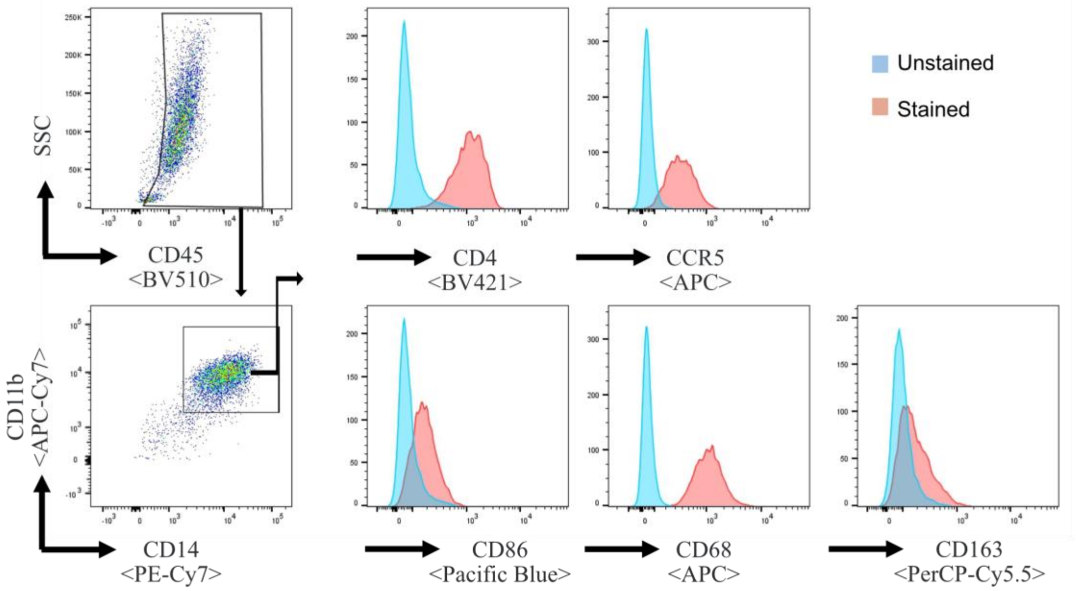

<u><R1887#1></u>

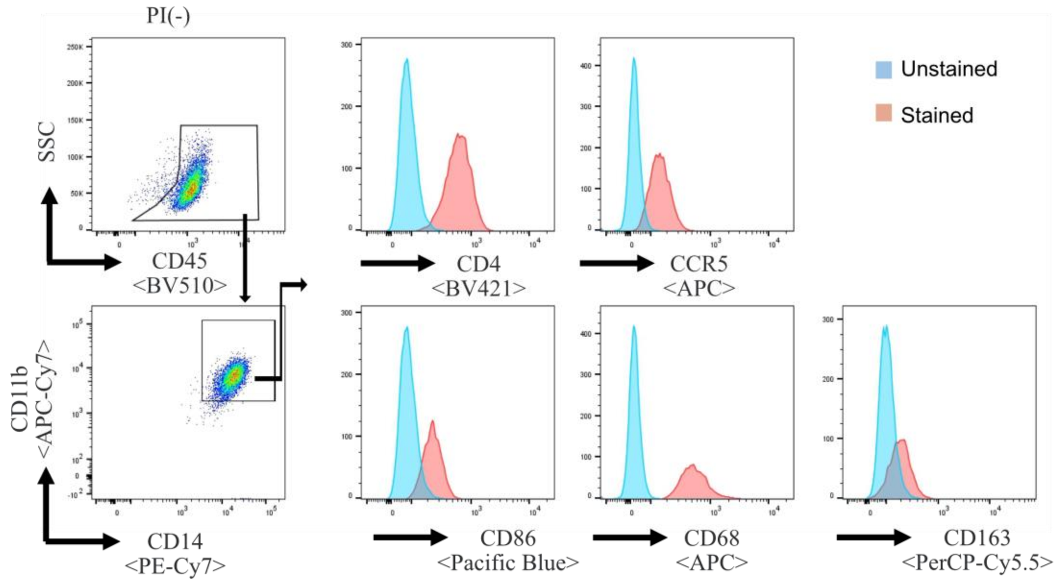

b

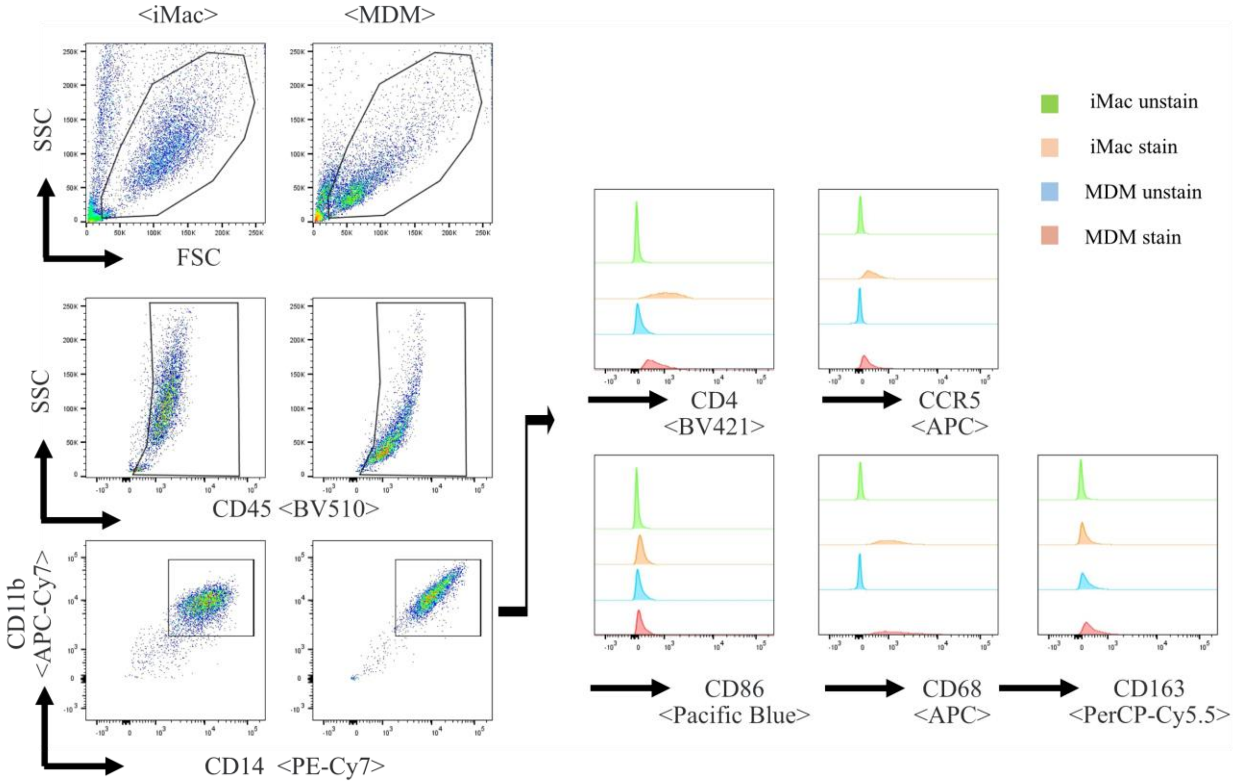

Figure S1 Flow-cytometric analysis of the macrophage differentiated from Rh-iPSC

(a)Flow-cytometric analysis of the macrophage generated from other Rh-iPSC clones phenotypes 34 days after the differentiation.

(b)Flow-cytometric analysis of macrophage-marker expression in macrophages generated from Rh-iPSCs and monocytes (MDM).

Table S1

Results of CRISPR/Cas9 genome editing by using Cas9 and gRNA expression plasmid (Exp1,2) or Cas9 protein and sgRNA(Exp3,4)

	Efficacy of genome editing
Exp1: Cas9 and gRNA expression plasmid	0%
Exp2: Cas9 and gRNA expression plasmid	0%
Exp3: Cas9 protein and sgRNA	29.1%
Exp4: Cas9 protein and sgRNA	40%
